# Acetylcholinesterase and Cytotoxic Activity of Chemical Constituents of *Clutia lanceolata* Leaves and its Molecular Docking Study

**DOI:** 10.1007/s13659-016-0110-x

**Published:** 2016-10-18

**Authors:** Mehtab Parveen, Faheem Ahmad, Ali Mohammed Malla, Shaista Azaz, Mahboob Alam, Omer A. Basudan, Manuela Ramos Silva, Pedro S. Pereira Silva

**Affiliations:** 1 Department of Chemistry, Aligarh Muslim University, Aligarh, 202002 India; 2 Division of Bioscience, Dongguk University, Gyeongju, 780-714 Republic of Korea; 3 Pharmacognosy, College of Pharmacy, King Saud University, Riyadh, 4451 Saudi Arabia; 4 CFisUC, Department of Physics, University of Coimbra, 3004-516 Coimbra, Portugal

**Keywords:** *Clutia lanceolata*, X-ray crystallography, AChE, Molecular docking, Cytotoxicity

## Abstract

**Abstract:**

Phytochemical investigations of the ethanolic extract of leaves of *Clutia lanceolata* (Family: Euphorbiaceae) resulted in the isolation of four compounds viz. 3,4-dihydroxy-2-methylbenzoic acid (**1**), 2,2′-dihydroxy-1,1′-binaphthyl (**2**), 1,3,8-trihydroxy-6-methylanthracene-9,10-dione (**3**) and 5-hydroxy-1,7-bis(4-hydroxy-3-methoxyphenyl)hepta-1,4,6-trien-3-one (**4**). Although all the isolated compounds were known but this was the first report from this plant source. Their structures were established on the basis of chemical and physical evidences viz. elemental analysis, FT-IR, ^1^H-NMR, ^13^C-NMR and mass spectral analysis. Structure of compound **2** and **4** was further authenticated by single-crystal X-ray analysis and density functional theory calculations. The isolated compounds (**1–4**) were screened for AChE enzyme inhibition assay in which compound **3** and **4** were found to be more potent AChE inhibitor. Molecular docking study of potent AChE inhibitor was performed to find the probable binding mode of the compounds into the active site of receptor. Moreover, the isolated compounds were also screened for in vivo cytotoxicity by brine shrimp lethality assay.

**Graphical Abstract:**

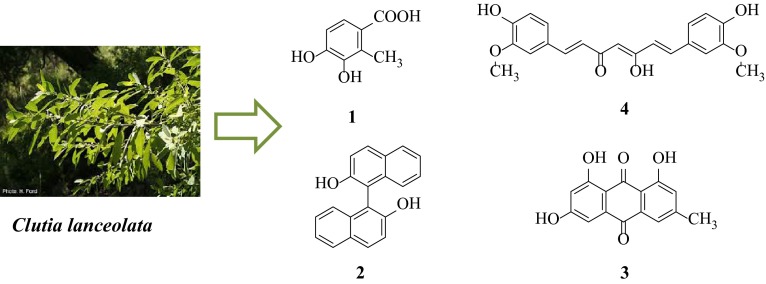

**Electronic supplementary material:**

The online version of this article (doi:10.1007/s13659-016-0110-x) contains supplementary material, which is available to authorized users.

## Introduction

**Fig. 1:**
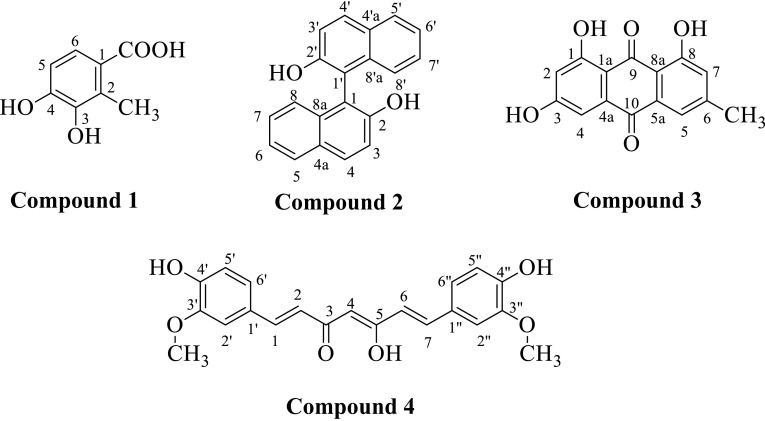
Structure of isolated compounds (**1**–**4**) from *Clutia lanceolata*

*Clutia* is a genus of plants belongs to the family Euphorbiaceae and is native to sub-Saharan Africa and the Arabian Peninsula. Ethnobotanical studies have uncovered several *Clutia* species which are widely used in folk medicine and have potential medicinal value [1]. The phytochemical constituents of the genus *Clutia* have not been investigated extensively, only few species have been explored phytochemically viz *C. richardiana* [2–7], *C. abyssinica* [8–10], *C. robusta* [11] and *C. similis* [12] etc. In this context, *Clutia lanceolata*, a woody herb or shrub or sometime grown up to tree belonging to the family Euphorbiaceae, is also relatively unexplored. Baka in 2010 showed the antifungal property of the aqueous extracts of its leaves [13] and Mossa et al. reported the hypoglycaemic property of *C. lanceolata* [14]. As part of our research work to explore the phytochemical and biological profile of medicinal plants [15, 16], we have carried out the phytochemical analysis of the leaves of *C. lanceolata*. The present paper reported the isolation, characterization, X-ray crystallographic study and biological activity of four compounds *i.e.* 3,4-dihydroxy-2-methylbenzoic acid (**1**), 2,2′-dihydroxy-1,1′-binaphthyl (**2**), 1,3,8-trihydroxy-6-methylanthracene-9,10-dione, Emodin (**3**) and 5-hydroxy-1,7-bis(4-hydroxy-3-methoxyphenyl)hepta-1,4,6-trien-3-one, curcumin (**4**), isolated from the ethanolic extract of *C. lanceolata* leaves (Fig. 1). The structure of all the isolated phytoconstituents was established on the basis of physical and chemical data (IR, ^1^H-NMR, ^13^C-NMR, and MS spectral analysis). However, the structure of compounds **2** and **4** was further authenticated with X-ray crystallographic analysis. To the best of our knowledge, these four compounds have not been reported from this plant source. Moreover, compound **2** was isolated from the only natural source *i.e.* the root of *Sesbania grandiflora* by Noviany et al. [17].

## Results and Discussion

### Structural Elucidation

Compound **1** was isolated as brick red crystalline solid, mp 233–235 °C. Elemental analysis along with the molecular ion peak at *m/z* 168.04 [M]^+**·**^ agreed with the molecular formula C_8_H_8_O_4_. The IR spectrum revealed characteristic absorption bands for hydroxyl group (3336 cm^−1^) and phenyl ring (1608 and 1489 cm^−1^). The presence of phenolic −OH group was further confirmed with the positive ferric chloride test. The IR spectrum also showed absorption bands at 3463 and 1685 cm^−1^ corresponded to carboxyl group which was further confirmed by the appearance of effervescences with NaHCO_3_. The ^1^H NMR spectrum indicated the presence of two *ortho* coupled doublets at *δ* 7.74 and 6.75 corresponding to the H-6 and H-5 aromatic protons, respectively. The downfield shift of proton H-6 is due to the presence of carboxylic group on adjacent carbon. The presence of two singlets at *δ* 10.06 and 9.67 integrating for two and one proton was corresponded to the two phenolic −OH and a carboxylic −OH, respectively. Moreover, a sharp singlet at *δ* 2.24 integrating for three protons indicates the presence of methyl group. The ^13^C NMR spectrum exhibited peaks at *δ* 13.75 and 178.43 which correspond to the presence of methyl and carbonyl carbon, respectively. Further, peaks at *δ* 148.0 (C-1), 128.3 (C-2), 158.60 (C-3), 158.69 (C-4), 127.98 (C-5) and 114.90 (C-6) showed the presence of six carbons of aromatic ring. In the light of above assignment and by comparing their spectral data and melting points with the reported literature [18], it was concluded that the compound **1** is ***3,4*** **-** ***dihydroxy*** **-** ***2*** **-** ***methylbenzoic acid***.

Compound **2** was obtained as white crystals, mp 207 °C with a molecular formula of C_20_H_14_O_2_ deduced from the positive ion ESIMS at *m/z* 286.10 [M]^+^. Elemental analysis was also in good agreement with the molecular formula C_20_H_14_O_2_. The UV spectrum of the compound exhibited characteristic absorption band for aromatic nucleus displaying *λ*_*max*_ at 227, 278 and 336 nm indicating the presence of a phenolic chromophore. The IR spectrum showed absorptions at 3425 cm^−1^ indicating −OH group, at 3028 cm^−1^ for aromatic C–H stretching and 1380–1615 cm^−1^ for C=C stretching of aromatic rings. The positive response of compound to ferric chloride specifies the phenolic nature of hydroxyl group. The ^1^H NMR spectrum displayed a singlet at *δ* 11.15 integrating for two protons corresponded to two hydroxyl group. Two independent *ortho*-coupled doublets with *J* = 9 Hz at *δ* 7.24 and 7.85 integrating for two protons each corresponded to the H-3/3′ and H-4/4′ protons of the A ring of naphthalene. The H-5/5′ and H-8/8′ protons of B ring displayed double doublets with *J* = 1.3 and 8.0 Hz at *δ* 7.94 and 7.12, respectively. There were two 3fold doublets at *δ* 7.38 and 7.44 corresponded to the remaining protons H-6/6′ and H-7/7′ of the naphthyl ring B exhibiting two *ortho*-couplings (*J* = 8.0 and 7.0 Hz) and one *meta*-coupling (*J* = 1.3 Hz) doublets, characteristics for the naphthyl ring. The ^13^C NMR spectrum displayed characteristic peaks of naphthyl ring integrating around at *δ* 118.4–133.9. However, the carbon of the connected naphthyl rings showed peaks at *δ* 110.8 and the carbon with hydroxyl group substitution displayed peak at *δ* 154.8. In the light of above data it was significantly concluded that compound **2** is ***2,2′*** **-** ***dihydroxy*** **-** ***1,1′*** **-** ***binaphthyl*** [17]. The structure of compound **2** was further authenticated with the X-ray crystallographic analysis.

Compound **3** was isolated as orange coloured solid with mp of 267–269 °C. Elemental analysis along with the molecular ion peak at *m/z* 270.05 [M]^+**·**^ agreed with the molecular formula C_15_H_10_O_5_. The compound responded positively to the ferric chloride test indicating the presence of phenolic –OH group, which further confirmed with the broad peak at 3510 cm^−1^ in the IR spectrum. The IR spectrum also displayed peak at 3056 and 2918 cm^−1^ corresponded to aromatic and aliphatic C–H stretching vibrations. However, peaks at 1676 and 1625 cm^−1^ corresponded to C=O and C=C bonds of *α,β*-unsaturated carbonyl functionality. The ^1^H NMR spectrum of compound showed four *meta*-coupled doublets at *δ* 7.81, 7.65, 7.30 and 7.09 corresponded to aromatic protons H-2, H-4, H-7 and H-5, respectively. Singlet at *δ* 12.13 and 12.02 integrating for two and one protons, respectively, corresponded to hydroxyl protons. Moreover, sharp singlet at *δ* 2.46 integrating for three protons was assigned to methyl protons. The ^13^C NMR spectrum exhibited peak for carbonyl carbons at *δ* 182.0 (C-9) and 192.5 (C-10), methyl carbon at *δ* 22.2, methyl substituted carbons at *δ* 149.3 (C-3) and for hydroxyl substituted carbons at *δ* 162.4 (C-1), 160.3 (C-6) and 162.6 (C-8). The spectrum also showed eight more peaks for aromatic carbons at *δ* 113.7 (C-8a), 121.3 (C-7), 119.9 (C-5), 133.8 (C-5a), 124.5 (C-4), 134.0 (C-4a), 136.9 (C-2) and 111.8 (C-1a). In the light of above data it was significantly concluded that compound **3** is ***1,3,8*** **-** ***trihydroxy*** **-** ***6*** **-** ***methylanthracene*** **-** ***9,10*** **-** ***dione*** (Emodin) [19].

Compound **4** was furnished as shining light orange coloured crystals with mp 177 °C. Elemental analysis along with the molecular ion peak at *m/z* 368.13 [M]^+**·**^ were in good agreement with the molecular formula C_21_H_20_O_6_. The IR spectrum showed characteristics peak for −OH at 3427 cm^−1^, aromatic and aliphatic C–H vibrations at 3065 and 2905 cm^−1^, respectively. The absorption peaks at 1682, 1480–1605 and 1120 cm^−1^ corresponded to C=O, C=C and C–O bands, respectively. The ^1^H NMR spectrum exhibited doublets at *δ* 7.58, 7.22, 6.65 and 6.51 corresponded to H-1, H-2, H-6 and H-7, respectively. However, a sharp singlet at 6.72 was assigned to H-4 proton. The broad singlet at *δ* 11.12 and 10.75 were assigned to phenolic and enolic hydroxy groups, respectively. The aromatic protons of compound **4** showed *ortho*-coupled doublets at *δ* 6.92, 6.81 and singlet at *δ* 7.10 integrating for two protons each corresponded to H-5′/5″, H-6′/6″ and H-2′/2″, respectively. The methoxy group showed singlet at *δ* 3.85 integrating for six protons. The ^13^C NMR spectrum exhibited peaks for aromatic carbons at *δ* 111.2, 116.5, 121.9, 127.8, 147.3 and 149.6 corresponded to C-2′/2″, C-5′/5″, C-6′/6″, C-1′/1″, C-3′/3″ and C-4′/4″, respectively. Moreover, the straight chain of compound showed peak at *δ* 142.3 and 124.8 for C-1 and C-2, 140.7 and 122.2 for C-7 and C-6, respectively. However, C-3, C-4 and C-5 displayed peaks at *δ* 182.4, 100.1 and 180.3, respectively. In light of the above discussion and with comparison of the data to the reported literature [20], it was concluded that compound **4** is ***5*** **-** ***hydroxy*** **-** ***1,7*** **-** ***bis(4*** **-** ***hydroxy*** **-** ***3*** **-** ***methoxyphenyl)hepta*** **-** ***1,4,6*** **-** ***trien*** **-** ***3*** **-** ***one*** *i.e.* Curcumin. The structure of compound **4** was also authenticated by X-ray crystallographic analysis.

### Crystal Structure of Compound **2** and **4**

**Fig. 2:**
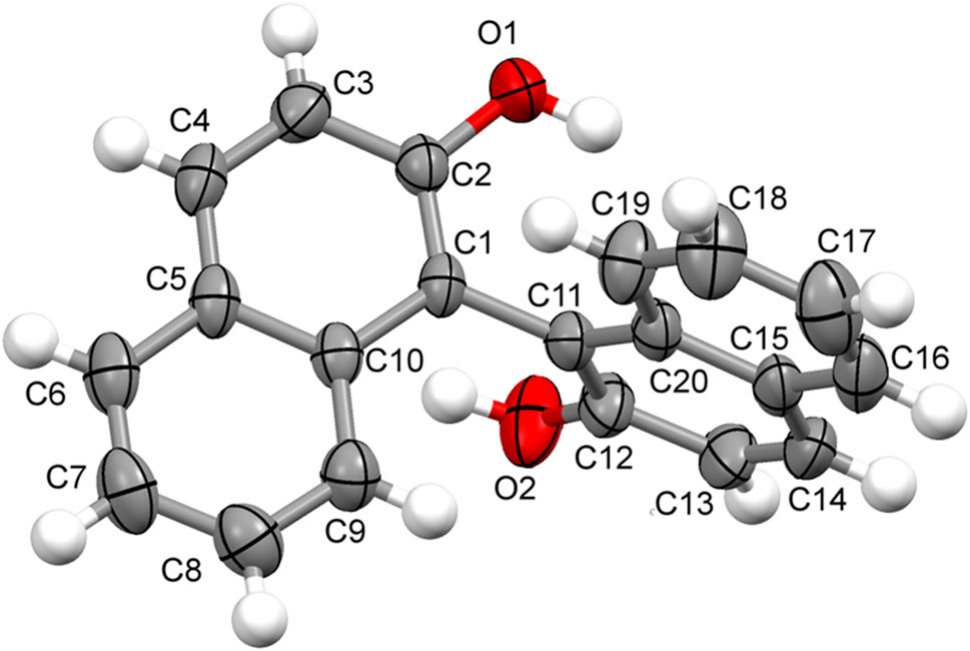
Asymmetric unit of compound **2** with the ellipsoids drawn at the 50 % probability level

**Fig. 3:**
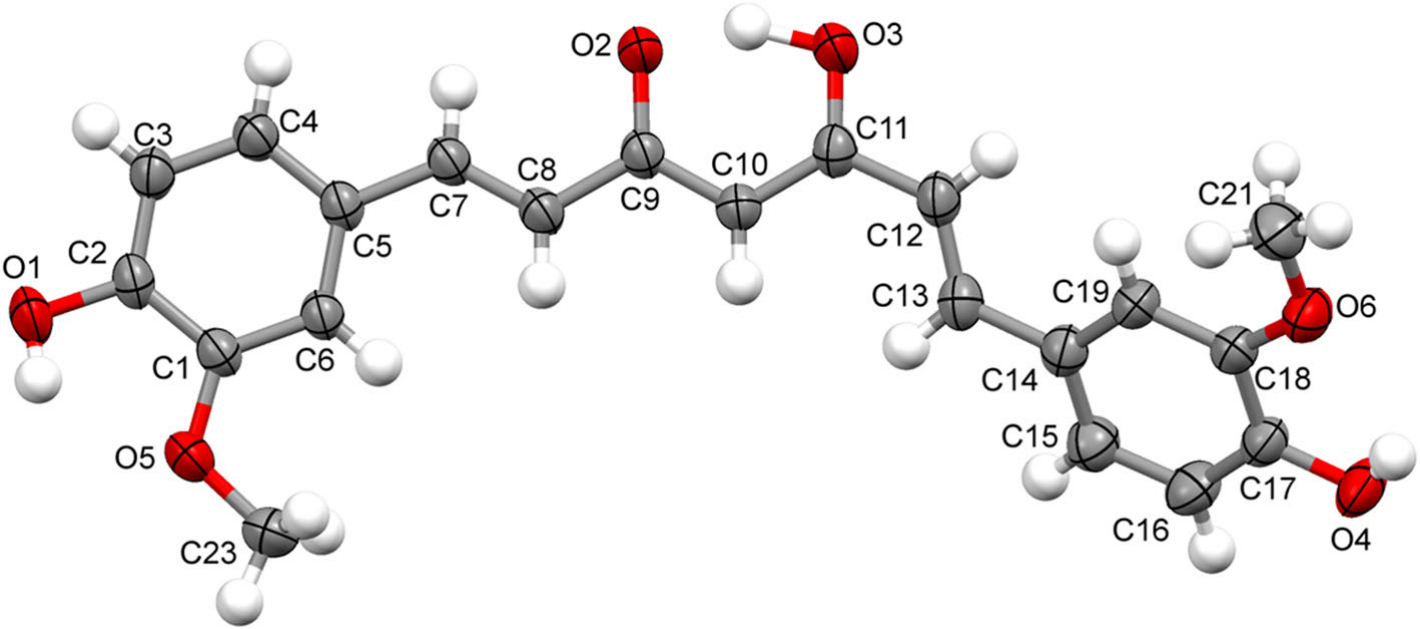
Asymmetric unit of compound **4** with the ellipsoids drawn at the 50 % probability level

Compound **2** and **4**, once isolated were found to be air-stable and soluble in all common organic solvents but insoluble in water. X-ray crystallographic analysis revealed that compound **2** crystallized in the orthorhombic structure with *Iba2* space group, while compound **4** crystallized in monoclinic crystal system with space group *P2/n*. These single crystal X-ray diffraction analysis of compounds **2** and **4** were in good agreement with the previous reports [21–23]. Asymmetric unit of compound **2** and **4** with the ellipsoids drawn at the 50 % probability level was shown in Figs. 2 and 3, respectively. The crystal data and structure refinement parameters were summarized in Table 1. The bond lengths and bond angles of compound **2** and **4** were presented in Tables 2 and 3. The crystal structure of compound **2** was stabilised by the intermolecular (O–H…O) hydrogen bonding. The bond length of O1-C2 and O2-C12 was 1.375 and 1.351 Å, respectively, showed partial double bond character due to the conjugation of lone pair of electrons of oxygen with naphthalene ring. The bond length between two naphthalene rings *i.e.* C1–C11 was 1.496 Å. The dihedral angle between two naphthalene rings C2-C1-C11 and C1-C11-C12 were 120.2(4)^o^ and 118.8(4)^o^, respectively. The crystal structure of compound **4** was stabilised by intramolecular (O–H…O) hydrogen bonding between O1H…O5, O4H…O6 and O3H…O2 and intermolecular (O–H…O and C–H…O) hydrogen bonds involving atoms O1H and H13 with atoms O3 and O5. The C–O bond lengths of O1-C2, O4-C17, O5-C1 and O6-C18 are in order of 1.36 Å less than typical single C–O bonds as the lone pair of electron of oxygen comes in conjugation with benzene ring and thus the C–O bonds attain partial double bond character. However, the bond length of O5-C23 and O6-C21 were in order of 1.41 Å and O2-C9 and O3-C11 had 1.29 and 1.28 Å, respectively. The C8–C9 and C11-C12 showed single bond character with bond length of 1.45 Å. However, C9–C10 and C10–C11 showed partial double band character with bond length of 1.38 and 1.40 Å. The angles of C8/C9/C10, C9/C10/C11 and C10/C11/C12 were 121.39(12)^o^, 120.62(12)^o^ and 124.30(13)^o^, respectively. The dihedral angles of C6/C5/C7/C8 and C12/C13/C14/C19 were −4.6(2)^o^ and 24.7(2)^o^, respectively.

**Table 1:** Crystal data and structure refinements of compound **2** and **4**

	Compound **2**	Compound **4**
Empirical formula	C_20_H_14_O_2_	C_21_H_20_O_6_
Formula weight	286.31	368.37
Temperature	293(2) K	293(2) K
Wavelength	0.71073 Å	0.71073 Å
Crystal system	Orthorhombic	Monoclinic
Space group	*Iba*2	*P*2/*n*
*a*	21.5986(8) Å	12.6851(5) Å
*b*	15.6844(6) Å	7.1848(3) Å
*c*	8.6243(3) Å	19.8951(8) Å
*α*	90º	90.00º
*β*	90º	95.223(2)º
*γ*	90º	90.00º
Volume	2921.58(19) Å^3^	1805.71(13) Å^3^
Z	8	4
Density (calculated)	1.302 gcm^−3^	1.355 gcm^−3^
Absorption coefficient	0.083 mm^−1^	0.099 mm^−1^
Extinction coefficient	0.0052(12)	–
*F*(000)	1200	776
Crystal size	0.53 × 0.48 × 0.24 mm^3^	0.60 × 0.46 × 0.44 mm^3^
θ range for data collection	3.11–25.84º	1.99–29.73º
Index ranges	−26 < *h* < 26; −19 < *k* < 19; −10 < *l* < 10	−17 < *h* < 13; −10 < *k* < 10; −27 < *l* < 27
Reflections collected	24389	39945
Independent reflections	2832	5076
Completeness to 2θ = 50º	99.8 %	98.8 %
Refinement method	Full matrix LS on *F* ^*2*^	Full matrix LS on *F* ^*2*^
Data/restrains/parameters	2832/1/206	5076/0/255
Goodness-of-fit on *F* ^*2*^	1.104	1.033
Final *R* indices [*I* > 2σ(*I*)]	*R* = 0.0487; *wR* = 0.1170	*R* = 0.0487; *wR* = 0.1408
*R* indices (all data)	*R* = 0.0781; *wR* = 0.1480	*R* = 0.0648; *wR* = 0.1570
Largest diff. peak and hole	0.163 and −0.204	0.342 and −0.301

**Table 2:** Comparison of selected geometrical parameters for compound **2** as determined by X-Ray diffraction and from the DFT geometry optimization (Å,°)

	Experimental	DFT
O1–C2	1.375(5)	1.358
O2–C12	1.351(6)	1.358
C1–C11	1.496(5)	1.496
C1–C2	1.367(6)	1.390
C11–C12	1.388(6)	1.390
C1–C2–C3	122.1(4)	121.4
C11–C12–C13	120.4(4)	121.3
C2–C1–C11	120.2(4)	118.9
C1–C11–C12	118.8(4)	118.9
C2–C1–C11–C12	−88.8(5)	−96.1
O1–C2–C3–C4	−180.0(4)	−180.0
O2–C12–C13–C14	−178.4(5)	−180.0

**Table 3:** Comparison of selected geometrical parameters for compound **4** as determined by X-Ray diffraction and from the DFT geometry optimization (Å,°)

	Experimental	DFT
O1–C2	1.3635(16)	1.3553
O4–C17	1.3583(16)	1.3552
O5–C1	1.3626(16)	1.3726
O6–C18	1.3606(18)	1.3717
O5–C23	1.4170(19)	1.4227
O6–C21	1.408(2)	1.4237
O2–C9	1.2982(17)	1.2554
O3–C11	1.2867(18)	1.3290
C8–C9	1.4578(17)	1.4721
C9–C10	1.3898(18)	1.4403
C10–C11	1.4052(18)	1.3793
C11–C12	1.4544(18)	1.4590
C1–O5–C23	117.42(11)	118.51
C18–O6–C21	118.19(12)	118.54
O5—C1–C6	125.72(13)	126.21
O6–C18–C19	125.18(13)	126.17
C8–C9–C10	121.39(12)	117.09
C9–C10–C11	120.62(12)	120.75
C10–C11–C12	124.30(13)	124.77
C23–O5–C1–C6	8.0(2)	0.5
C21–O6–C18–C19	5.3(2)	2.1
C6–C5–C7–C8	−4.6(2)	0.0
C12–C13–C14–C19	24.7(2)	13.9

### DFT Results of Compounds **2** and **4**

**Fig. 4:**
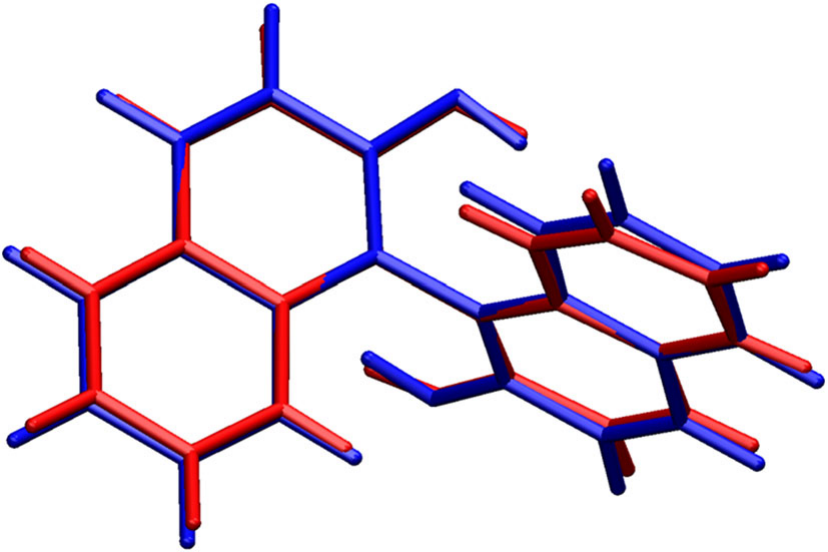
Comparison of the molecular conformation of compound **2**, as established from the X-ray study (*red*) with the optimized geometry (*blue*). (Software used for visualization: VMD, version 1.9.1, January 29, 2012 [24])

**Fig. 5:**
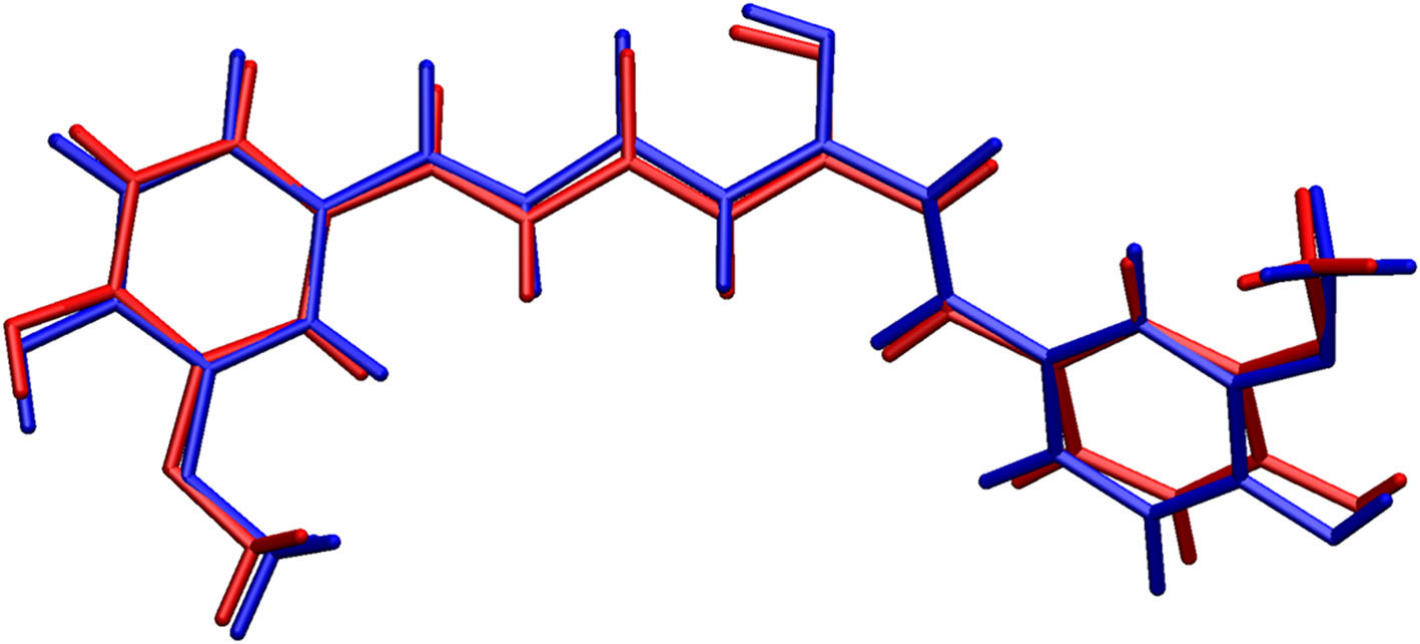
Comparison of the molecular conformation of compound **4**, as established from the X-ray study (*red*) with the optimized geometry (*blue*). (Software used for visualization: VMD, version 1.9.1, January 29, 2012 [24])

To examine the influence of the intermolecular interactions on the molecular geometries we have performed DFT calculations of the equilibrium geometries of the free molecules starting with the experimental X-ray geometries. For the compound **2**, there was a good agreement between the experimental and calculated geometries (Table 2; Fig. 4) with the largest difference occurring in the torsion angle C2-C1-C11-C12 (7.3º), however this was not unexpected, since the rotation around a C–C single bond did not have a high energy cost [24]. The situation was quite different for compound **4**. In the experimental geometry, there was a high degree of chemical symmetrization in the enol ring, defined as near equality of the C–C and C–O bond lengths compared pairwise, according to Herbstein et al. [25]. The theoretical geometry did not display this symmetrization. One possible explanation for this discrepancy may be the O–H…O hydrogen bonds involving the oxygen atoms of the enol ring and hydroxyl groups, that linked different curcumin molecules (Table 3; Fig. 5).

### AChE Inhibition Results

The acetylcholinesterase inhibition effect of the isolated compounds (**1**–**4**) was assessed by Ellman’s spectrophotometric method using human recombinant AChE with tacrine as reference compound. The result obtained has been summarized in Table 4. It can be inferred from the data shown in Table 4, that compound **3**, exhibited the strongest inhibition to AChE with an IC_50_ value of 14.2 µM, followed by compound **4** (IC_50_ = 16.4 µM), **2** (IC_50_ = 21.9 µM) and **1** (IC_50_ = 23.7 µM). The results indicated that all the isolated compounds displayed moderate inhibitory activity against the acetylcholinesterase enzyme. However, compounds **3** and **4** were found to be more active in comparison to **1** and **2**. This was mainly attributed to the basic skeleton of compound **3** and **4** which provide them more binding prospects with the formation of hydrogen bonding interactions to the amino acid residues of the protein. However, the π–π stacking may also leads to improve its activity with the formation of additional non-bonding interactions to the protein.

**Table 4:** In vitro AChE inhibition IC_50_ (μM) of compounds **1**–**4** and reference drug tacrine

S. no.	Compounds	IC_50_ (µM)^a^ ± SEM for *h*AChE inhibition
1	Compound **1**	23.7 ± 0.02
2	Compound **2**	21.9 ± 0.05
3	Compound **3**	14.2 ± 0.06
4	Compound **4**	16.4 ± 0.03
5	Tacrine (standard)	0.20 ± 0.01

^**a**^IC_50_ = Concentration of inhibitor required to decrease enzyme activity by 50 %

### Molecular Docking Studies

**Fig. 6:**
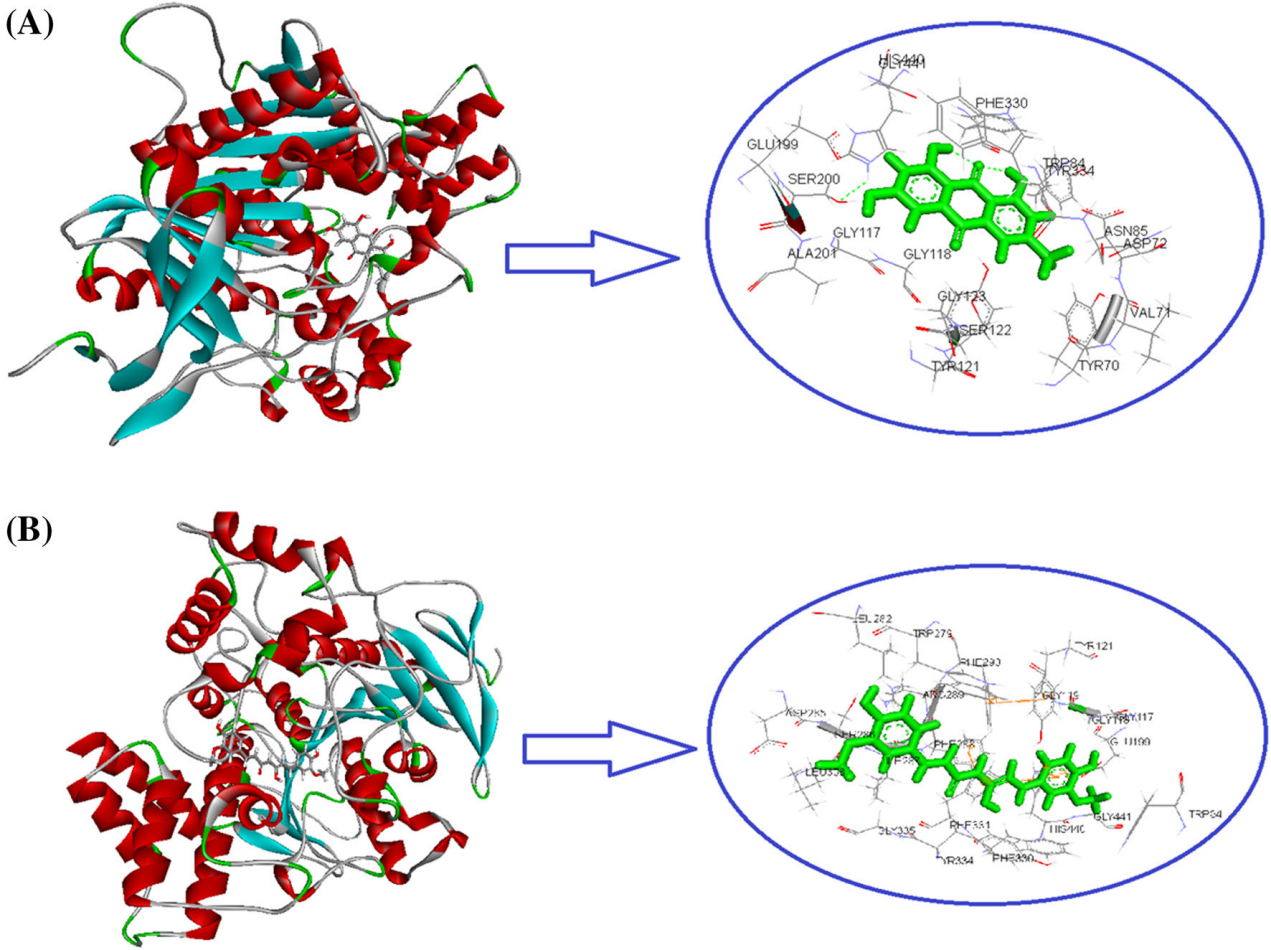
Interactions profile of **a** compound **3** and **b** compound **4** with receptor

**Fig. 7:**
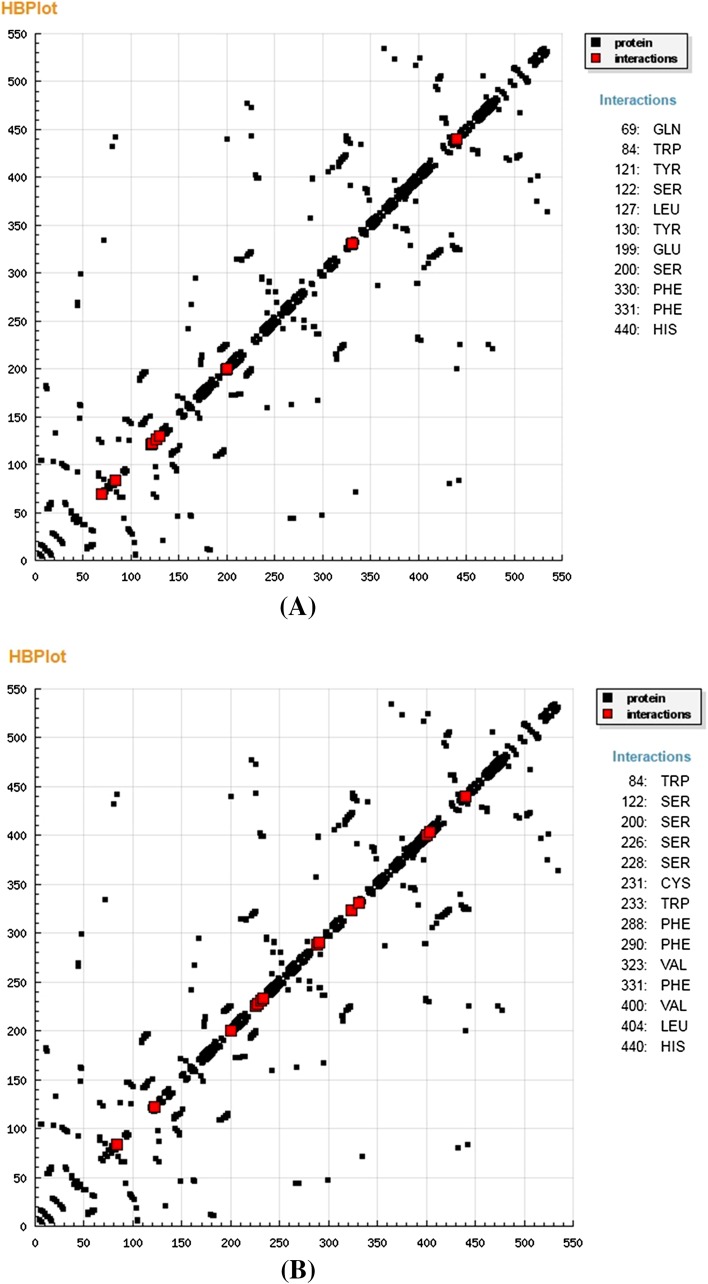
HB plot of interacted residues in protein with compound **3** (**a**) and compound **4** (**b**)

The molecular docking studies were conducted in order to get insight the binding pattern and extent of binding of compounds with the target enzymes. In the present study, we have carried out the docking study of the two most potent acetylcholinestrerase inhibitor *i.e.* compounds **3** and **4** of the isolated compounds in order to anticipate the binding mode towards target enzyme (PDB: 1EVE) and to give justification for the observed in vitro AChE inhibition property of the isolated compounds **3** and **4**. The in silico docking experiment for compounds **3** and **4** against the X-ray crystal structure of receptor (PDB: 1EVE), was carried out using PATCHDOCK and iGEMDOCK software. Docking simulations showed comparable binding affinity of compounds **3** and **4** with AChE enzyme. Several interactions from the docking pose were observed notably TYR121; SER122; LEU127; TYR130; GLU199; SER200; PHE330; PHE331 and HIS440 between the receptor and compound **3**; SER 122; SER226; CYS231; TRP233; PHE288; VAL323; LEU404 and HIS440 between the receptor and compound **4** in proper binding orientations. The binding score from iGEMDOCK was found to be −108.18 and −98.91 kcal/mol for compounds **3** and **4**, respectively. This is due to the cumulative van der Wall contribution and H-bonding interactions. The docking studies revealed that the aromatic ring also plays a major role in stabilizing the ligand-receptor complex by *pi*-cation interactions with amino acid residue of the target protein as shown in Fig. 6. These strong interactions help the compounds to bury well inside the cavity of target protein and acts as a potent AChE inhibitor. The analysis of enzyme inhibition data obtained from the in vitro experiments, showed the comparative inhibitory property of compound **3** (IC_50_ = 14.2 µM) and compound **4** (IC_50_ = 16.4 µM) and found to be moderately potent compared to a standard drug tacrine (IC_50_ = 0.20 µM), as AChE inhibitor. The HB plot of the interacted residues in protein of AChE with compounds **3** and **4** was depicted in Fig. 7. This plot helped us to study the way protein residues interacts with ligand.

### Cytotoxicity Studies

The in vivo cytotoxicity of compounds (**1–4**) isolated from the leaves of *C. lanceolata* was evaluated to brine shrimp nauplii using vincristine sulfate as standard (Fig. S1 †ESI). It is one of the most convenient methods to determine the cytotoxicity of any compound. The LD_50_ value of compounds was reported in ppm units and summarized in Table 5. In the present study the LD_50_ value of the compounds were found to be 23.40 µg/mL (compound **1**), 17.78 µg/mL (compound **2**), 13.80 µg/mL (compound **3**) and 11.66 µg/mL (compound **4**) as compared to the standard drug vincristine sulfate whose LD_50_ was found to be 8.84 µg/mL. These data clearly signified the considerable cytotoxicity of all the isolated compounds (**1**–**4**). However, compound **4** showed most lethality to the brine shrimp than the other isolated compounds **1**–**3**, almost comparable to the standard drug.

**Table 5:** In vivo cytotoxicity assay of isolated compounds (**1**–**4**)

S. no.	Compound	LD_50_ value (μg/mL)
1	Compound **1**	23.40 ± 0.02
2	Compound **2**	17.78 ± 0.05
3	Compound **3**	13.80 ± 0.03
4	Compound **4**	11.66 ± 0.04
5	Vincristine sulphate (Standard)	8.84 ± 0.01

## Experimental Section

### General Experimental Procedures

All solvents and chemicals were purchased from commercial sources (Sigma-Aldrich, Merck, and others) and used as received or dried using standard procedures. Melting points were determined on a Kofler apparatus and are uncorrected. Elemental analysis (CHN) has been conducted using a Thermo Scientific (FLASH 2000) CHN Elemental Analyser. Fourier transform-infrared (FT-IR) spectra were recorded using a Perkin-Elmer (2000 FTIR) Spectrometer by the KBr pellet method, values are given in cm^−1^. The UV spectra were recorded with PerkinElmer UV Win Lab spectrophotometer. ^1^H and ^13^C NMR spectra were run in CDCl_3_ and DMSO-*d*_*6*_ on Bruker Avance-II 400 and 100 MHz instruments, respectively. Mass spectra were recorded on a JEOL D-300 mass spectrometer. Thin-layer chromatography (TLC) glass plates were coated with silica gel (E-Merck G254) and exposed to iodine vapour to check the purity of the isolated compounds.

### Plant Materials

The leaves of *C. lanceolata* were collected from Abha region of South of Saudi Arabia and identified by Dr. S. Adeen, Taxonomist of the Medicinal, Aromatic and Poisonous Plant Research Centre (MAPPRC), College of Pharmacy, King Saud University, Riyadh, Saudi Arabia. A voucher specimen bearing number 14077 has been deposited in their herbarium.

### Extraction and Isolation

The air-dried leaves of *C. lanceolata* were crushed to make powder (2.0 kg) and extracted exhaustively with 95 % ethanol about three times under reflux temperature and filtered to yield a filtrate. The solvent was evaporated under reduced pressure to afford a crude extract and fractionated successively with petroleum ether, benzene, ethyl acetate and methanol. The petroleum ether and benzene extracts showed similar behaviour on TLC hence were mixed together. The mixed petroleum ether and benzene extract was chromatographed on a silica gel column, eluting stepwise with petroleum ether-benzene (1:0, 9:1 → 1:9) which afforded compounds **1** and **2**. Similarly, the ethyl acetate extracts was subjected to column chromatography on silica gel, eluting stepwise with benzene-ethyl acetate (1:0, 9:1 → 1:9, 0:1) which furnished two compounds i.e. compounds **3** and **4**. The isolated compounds were purified by repeated column chromatography followed by crystallization to get pure compounds.

### Spectral Characterization of Isolated Compounds

#### 3,4-Dihydroxy-2-methylbenzoic acid (1)

Brick red crystalline solid; mp 233–235 °C; IR (KBr) υ_max_ 3463, 3336, 3184, 1685, 1608, 1489, 1431, 1264, 1228, 1173, 1089, 827 cm^−1^; ^1^H NMR (DMSO d_6_, 400 MHz) *δ* 10.06 (2H, s, 2 × –OH), 9.67 (1H, s, −COOH), 7.74 (1H, d, *J* = 8.0 Hz, H-6), 6.75 (1H, d, *J* = 8.0 Hz, H-5), 2.24 (3H, s, –CH_3_); ^13^C NMR (DMSO d_6_,100 MHz) *δ* 178.4 (C, COOH), 158.6 (C, C-3), 158.6 (C, C-4), 148.0 (C, C-1), 128.3 (C, C-2), 127.9 (CH, C-5), 114.9 (CH, C-6), 13.7 (–CH_3_); ESIMS *m/z* 168.04 [M]^+·^ (C_8_H_8_O_4_); Anal. Calc. for C_8_H_8_O_4_: C, 57.14; H, 4.80; found: C, 58.09; H, 4.95.

#### 2,2′-Dihydroxy-1,1′-binaphthyl (2)

White crystals; mp 207 °C; UV (MeOH) λ_max_ 227, 278, 336 nm; IR (KBr) υ_max_ 3425, 3382, 2971, 2918, 2840, 1615, 1590, 1461, 1380, 1216, 1175, 1146, 826, 750 cm^−1^; ^1^H NMR (CDCl_3_, 400 MHz) *δ* 11.15 (2H, s, 2 × OH), 7.94 (2H, dd, *J* = 1.3 and 8.0 Hz, H-5 and H-5′), 7.85 (2H, d, *J* = 9.0 Hz, H-4 and H-4′), 7.44 (2H, m, H-7 and H-7′), 7.38 (2H, m, H-6 and H-6′), 7.24 (2H, d, *J* = 9.0 Hz, H-3 and H-3′), 7.12 (2H, dd, *J* = 1.3 and 8.0 Hz, H-8 and H-8′); ^13^C NMR (CDCl_3_, 100 MHz) *δ* 154.3 (C, C-2 and C-2′), 133.9 (C, C-8a and C-8′a), 130.0 (CH, C-4 and C-4′), 129.2 (C, C-4a and C-4′a), 128.2 (CH, C-5 and C-5′), 126.9 (CH, C-7 and C-7′), 125.7 (CH, C-6 and C-6′), 123.5 (CH, C-8 and C-8′), 118.4 (CH, C-3 and C-3′), 110.8 (C, C-1 and C-1′); ESIMS *m/z* 286.10 [M]^+**·**^ (C_20_H_14_O_2_); Anal. Calc. for C_20_H_14_O_2_: C, 83.90; H, 4.93; found: C, 84.88; H, 5.05.

#### 1,3,8-Trihydroxy-6-methylanthracene-9,10-dione (3)

Orange coloured crystals; mp 267–269 °C; IR (KBr) υ_max_ 3510, 3056, 2918, 1676, 1625, 1565, 1458, 1371, 1272, 1205, 1161, 1082, 1026, 749 cm^−1^; ^1^H NMR (CDCl_3_, 400 MHz) *δ* 12.13 (2H, s, 2 × OH), 12.02 (1H, s, OH), 7.81 (1H, d, *J* = 1.2 Hz, H-2), 7.65 (1H, d, *J* = 1.2 Hz, H-4), 7.30 (1H, d, *J* = 1.2 Hz, H-7), 7.09 (1H, d, *J* = 1.2 Hz, H-5), 2.46 (3H, s, –CH_3_); ^13^C NMR (CDCl_3_, 100 MHz) *δ* 192.5 (C = O, C-10), 182.0 (C = O, C-9), 162.6 (C, C-8), 162.4 (C, C-1), 160.3 (C, C-6), 149.3 (C, C-3), 136.9 (CH, C-2), 134.0 (C, C-4a), 133.8 (C, C-5a), 124.5 (CH, C-4), 121.3 (CH, C-7), 119.9 (CH, C-5), 113.7 (C, C-8a), 111.8 (C, C-1a), 22.2 (CH_3_); ESIMS *m/z* 270.05 [M]^+**·**^ (C_15_H_10_O_5_); Anal. Calc. for C_15_H_10_O_5_: C, 66.67; H, 3.73; found: C, 67.58; H, 3.85.

#### 5-Hydroxy-1,7-bis (4-hydroxy-3-methoxyphenyl)hepta-1,4,6-trien-3-one (4)

Yellow-orange coloured crystals; mp 177 °C; IR (KBr) υ_max_ 3427, 3065, 2905, 1682, 1605, 1480, 1120 cm^−1^; ^1^H NMR (CDCl_3_, 400 MHz) *δ* 11.12 (2H, s, 2 × -OH), 10.75 (1H, s, -OH), 7.58 (1H, d, H-1), 7.22 (1H, d, H-2), 7.10 (2H, s, H-2′ and H-2″), 6.92 (2H, d, *J* = 8.0 Hz, H-5′ and H-5″), 6.81 (2H, d, *J* = 8.0 Hz, H-6′ and H-6″), 6.72 (1H, s, H-4), 6.65 (1H, d, H-6), 6.51 (1H, d, H-7), 3.85 (6H, s, 2 × -OCH_3_); ^13^C NMR (CDCl_3_, 100 MHz) *δ* 182.4 (C, C-3), 180.3 (C, C-5), 149.6 (C, C-4′ and C-4″), 147.3 (C, C-3′ and C-3″), 142.3 (CH, C-1), 140.7 (CH, C-7), 127.8 (C, C-1′ and C-1″), 124.8 (CH, C-2), 122.2 (CH, C-6), 121.9 (CH, C-6′ and C-6″), 116.5 (CH, C-5′ and C-5″), 111.2 (CH, C-2′ and C-2″), 100.1 (CH, C-4), 55.6 (2 × -OCH_3_); ESIMS *m/z* 368.13 [M]^+**·**^ (C_21_H_20_O_6_); Anal. Calc. for C_21_H_20_O_6_: C, 68.47; H, 5.47; found: C, 69.81; H, 5.85.

### X-ray Crystallographic Analysis

The crystal structures of the compounds **2** and **4** were determined by X-ray diffraction experiments performed on a Bruker Apex II diffractometer. The diffraction data was collected at room temperature 293(2) K using graphite monochromated Mo K*α* (*λ* = 0.71073 Å). Data reduction was performed with APEX II [26]. Lorentz and polarization corrections were applied. Absorption corrections were applied using SADABS [27]. An extinction correction was applied during the refinement of the crystal structure of compound **2** [28]. The crystallographic structures were solved using direct methods (SHELXS-97) [28]. The structure refinements were carried out with SHELXL-97 software [28]. The refinements were made by full-matrix least-squares on *F* ^*2*^, with anisotropic displacement parameters for all non-hydrogen atoms. All the hydrogen atoms were located in a difference Fourier synthesis, placed at calculated positions and then, included in the structure factor calculation in a riding model using SHELXL-97 defaults with the exception of the hydrogen atoms bonded to hetero atoms, which were refined freely. MERCURY 3.3 [29] was used for figure plotting. PLATON [30] was used for data analysis. Additional information to the structures determination is given in Table 1. Atomic coordinates, thermal parameters and bond lengths and angles have been deposited at the Cambridge Crystallographic Data Centre (CCDC) with reference numbers 1463766 and 1463767.

### DFT Calculations

The geometry optimizations were performed using the GAMESS package [31], starting from the experimental X-ray geometries. The calculations were performed within density functional theory (DFT) using B3LYP (Becke three-parameter Lee–Yang–Parr) for exchange and correlation, which combines the hybrid exchange functional of Becke [32, 33] with the correlation functional of Lee, Yang and Parr [34]. The calculations were performed with an extended 6-311G(d, p) basis set. Tight conditions for convergence of both the self-consistent field cycles and the maximum density and energy gradient variations were imposed (10^−5^ atomic units). At the end of each geometry optimization we conducted a Hessian calculation to guarantee that the final structure corresponds to a true minimum, using the same level of theory as in the geometry optimization.

### In Vitro AChE Inhibition Study

All the isolated compounds (**1–4**) were assessed for AChE inhibition study by Ellman’s method [35, 36]. AChE stock solution was prepared by dissolving human recombinant AChE (EC: 3.1.1.7) lyophilized powder (Sigma-Aldrich) in 0.1 M phosphate buffer (*p*H = 8.0) containing Triton X-100 (0.1 %). Five increasing concentrations of test compounds were assayed to obtain  % inhibition of the enzymatic activity in the range of 20–80. The assay solution consisted of a 0.1 M phosphate buffer *p*H 8.0, with the addition of 340 µM 5,50-dithio-bis(2-nitrobenzoic acid), 0.02 unit per mL of human recombinant AChE from human serum and 550 µM of the substrate (acetylthiocholine iodide, ATCh). Increasing concentrations of the tested inhibitor were added to the assay solution and pre-incubated for 5 min at 37 °C with the enzyme followed by the addition of the substrate. Initial rate assays were performed at 37 °C using a Jasco V-530 double beam Spectrophotometer. The absorbance value at 412 nm was recorded for 5 min and enzyme activity was calculated from the slope of the obtained linear trend. Assays were carried out with a blank containing all components except AChE to account for the non-enzymatic reaction. The reaction rates were compared and the percent inhibition due to the presence of tested inhibitors was calculated. Each concentration was analysed in triplicate, and IC_50_ values were determined graphically from log concentration-inhibition curves (GraphPad Prism 4.03 software, GraphPad Software Inc.). Tacrine was used as a standard inhibitor.

### Molecular Docking

Docking simulations of compounds **3** and **4** were carried out according to the method described previously [37, 38]. The energy minimized structure of compounds **3** and **4** was sketched with ChemDraw Ultra (2D and 3D). The coordinates of compounds **3** and **4** was checked using PRODRG program [39] for generating molecular topologies. The three-dimensional structures of target protein function as a receptor (PDB: 1EVE) and was retrieved from the protein data bank. All the heteroatoms coupled with proteins including water molecules, bound ligands and any co-crystallized solvent were discarded from the PDB file and the missing assignments like proper bonds, bond orders, hybridization and charges were assigned using the Molegro Virtual Viewer [40]. PATCHDOCK [41], iGEMDOCK [42] and Acceryl Discovery Studio 4.0 Client [43] were employed to evaluate the molecular docking, energy profile and visualization of compounds-receptor interactions, respectively.

### In Vivo Cytotoxicity Assay

Brine shrimp lethality bioassay is commonly used in the bioassay for the bioactive compounds [44, 45]. The in vivo cytotoxicity assay was performed on brine shrimp nauplii (*Artemia salina*) in accordance with the Mayer method [46]. The egg of brine shrimp were hatched in a tank filled with artificially prepared sea water (brine, 3.8 % NaCl) exposed to incandescent light. Air was supplied at the bottom of the tank with the help of air supplier fitted with tube to keep the shrimps in uniform motion. The assay was performed 24 h after hatching and no food supplement was given during the hatching and experimental periods. A test sample (5.0 mg) was dissolved in 1 mL DMSO to obtain stock solution of 5 mg/mL. Different concentrations of test samples were obtained from stock solution and placed in separate vials, and the volume of each vial was made up to 5 mL with brine to obtain the desired final concentrations (10, 20, 40, 60 and 80 µg/mL). The negative control was prepared in the same manner without the samples. Vincristine sulphate was used as a standard anti-cancer drug. Thirty brine shrimp nauplii were then placed in each vial. After 24 h of incubation, the vials were observed using a magnifying glass, and the number of survivors in each vial were counted and noted. The LD_50_ values were calculated using the plot of percentage of mortality and logarithm of concentration. All tests were performed in triplicate and expressed as mean.

## Conclusion

This is the first report of the isolation of these four compounds from *Clutia lanceolata* leaves viz. 3,4-dihydroxy-2-methylbenzoic acid (**1**), 2,2′-dihydroxy-1,1′-binaphthyl (**2**), 1,3,8-trihydroxy-6-methylanthracene-9,10-dione (**3**) and 5-hydroxy-1,7-bis(4-hydroxy-3-methoxyphenyl)hepta-1,4,6-trien-3-one (**4**). Molecular structure of compounds **2** and **4** was authenticated unambiguously by X-ray crystallography and DFT studies. All the isolated compounds (**1**–**4**) showed significant activity for cytotoxicity and AChE inhibition assay. Compound **4** showed significant lethality to the brine shrimp nauplii. As far as AChE inhibition concerned, compounds **3** and **4** acted as potent enzyme inhibitors. Molecular docking study validated the binding pattern and extent of binding of compounds **3** and **4** with the target enzymes.

## Electronic supplementary material

Below is the link to the electronic supplementary material.

Supplementary material 1 (DOCX 176 kb)
