# Supplementary material for: Acetylcholinesterase and Cytotoxic Activity of Chemical Constituents of Clutia lanceolata Leaves and its Molecular Docking Study
Source: Nat Prod Bioprospect. 2016 Oct 18;6(6):267–78. doi: 10.1007/s13659-016-0110-x (PMC5136371; doi:10.1007/s13659-016-0110-x)
Supplement: Supplementary file 1 — Supplementary material 1 (DOCX 176 kb) [file 13659_2016_110_MOESM1_ESM.docx]

**Supplementary Information**

**Acetylcholinesterase and Cytotoxic Activity of Chemical Constituents of *Clutia lanceolata* Leaves and its Molecular Docking Study**

Mehtab Parveen,*^a^ Faheem Ahmad,^a^ Ali Mohammed Malla,^a^ Shaista Azaz,^a^ Mahboob Alam,^b^ Omer A. Basodan,^c^ Manuela Ramos Silva^d^ and Pedro S. Pereira Silva^d^

*^a^Department of Chemistry, Aligarh Muslim University, Aligarh, 202002, India*

*^b^Division of Bioscience, Dongguk University, Gyeongju 780-714, Republic of Korea*

*^c^Pharmacognosy, College of Pharmacy, King Saud University, Riyadh 4451, Saudi Arabia*

*^d^CFisUC, Department of Physics, University of Coimbra, 3004-516 Coimbra, Portugal*

**Corresponding author: Mehtab Parveen*

*E-mail: mehtab.organic2009@gmail.com; Tel: +91-9897179498*

**Fig. S1** Determination of LD_50_ values for compounds (**1**-**4**) from linear correlation between logarithms of concentration *versus* percentage of mortality.

*
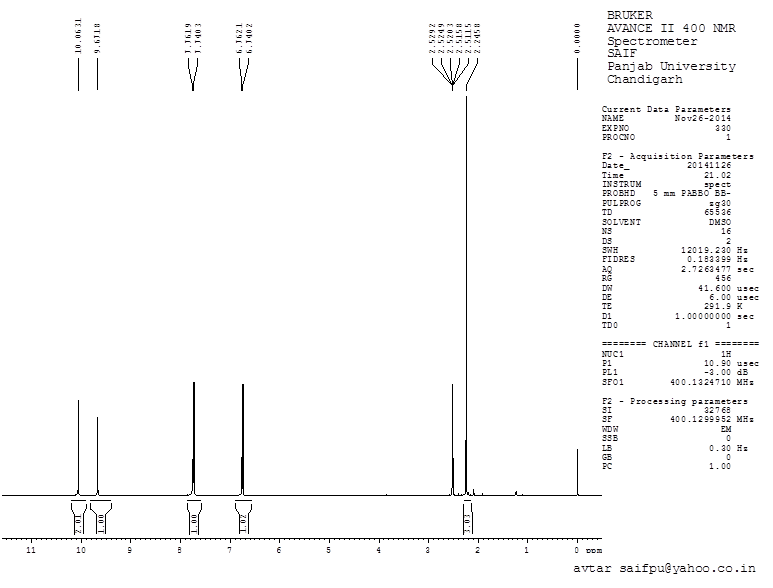
*

**^1^H-NMR spectrum of Compound 1**

*
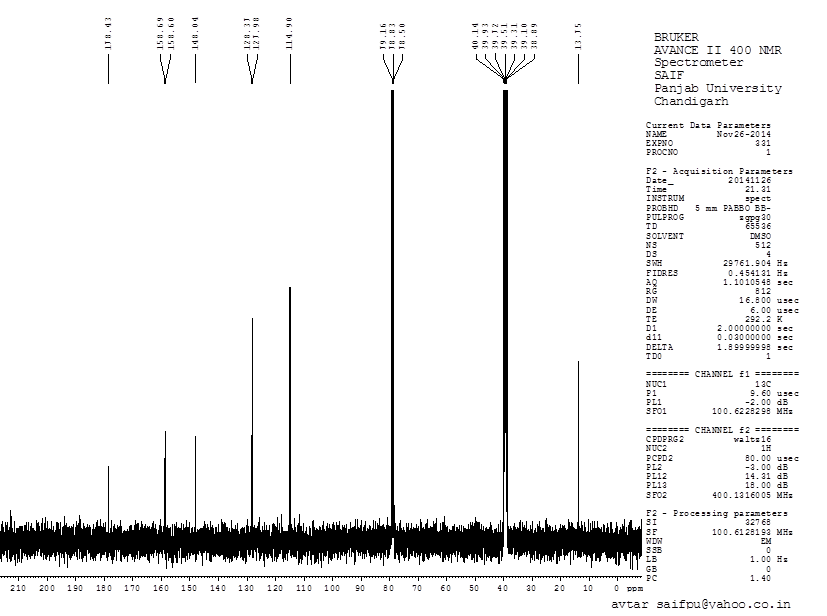
*

**^13^C-NMR spectrum of Compound 1**

*
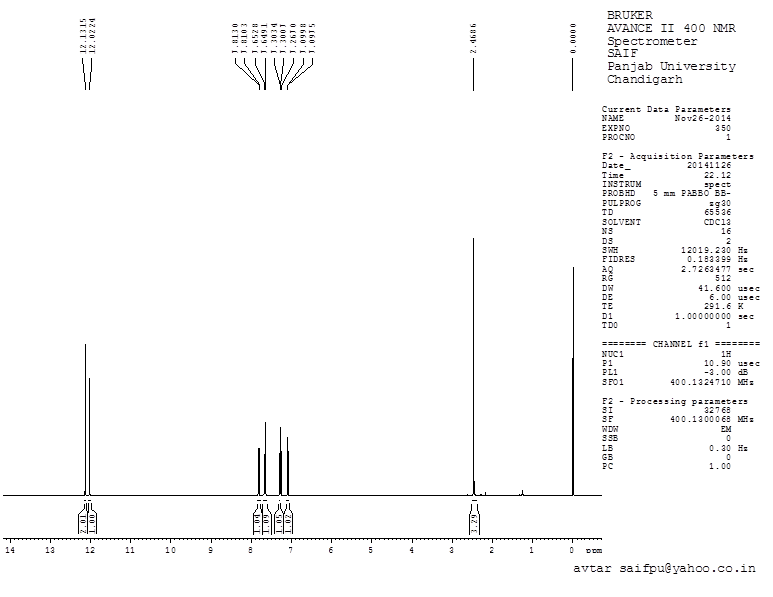
*

**^1^H-NMR spectrum of Compound 3**

*
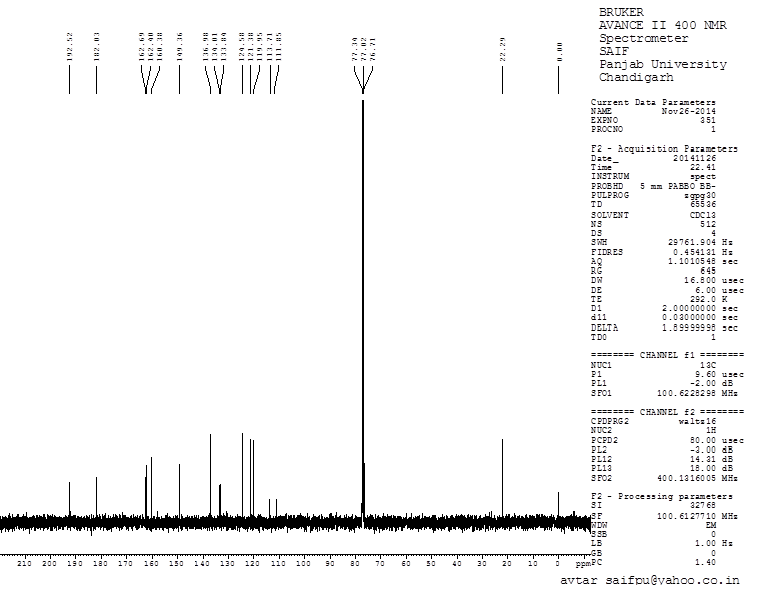
*

**^13^C-NMR spectrum of Compound 3**
